# Dietary Administration of Novel Multistrain Probiotics from Healthy Grouper Intestines Promotes the Intestinal Immune Response against NNV Infection

**DOI:** 10.3390/life11101053

**Published:** 2021-10-07

**Authors:** Joan Tang Xiao Joe, Henry Tan Shi Sung, Jen-Leih Wu, Yu-Shen Lai, Ming-Wei Lu

**Affiliations:** 1 Department of Aquaculture, National Taiwan Ocean University, Keelung 20224, Taiwan; 20708002@mail.ntou.edu.tw (J.T.X.J.); 00633043@mail.ntou.edu.tw (H.T.S.S.); 2 Institute of Cellular and Organismic Biology, Academia Sinica, Taipei City 11529, Taiwan; jlwu@gate.sinica.edu.tw; 3 Institute of Biotechnology, National Ilan University, Ilan 26047, Taiwan; yslai@niu.edu.tw; 4 Center of Excellence for the Oceans, National Taiwan Ocean University, Keelung 20224, Taiwan

**Keywords:** grouper, nervous necrosis virus, intestine, innate immunity, adaptive immunity

## Abstract

*Epinephelus lanceolatus* (giant grouper) is a high-value cultured species in the Asia-Pacific region. However, nervous necrosis virus (NNV) is an infectious viral disease that affects over 120 species of marine cultured species and causes high mortality, ranging from 90–100% in the grouper industry. Probiotics isolated from the intestines of healthy individuals have provided insight into novel approaches involved in the defense against viral pathogens. In this study, we isolated three strains of bacteria as candidate probiotics from healthy grouper intestines and a 28-day feeding trial was performed. At day 21, the nervous necrosis virus (NNV) challenge test was conducted for 7 days to evaluate the antiviral effect of candidate probiotics. The results showed that candidate probiotics could improve growth conditions, such as weight gain (WG) and specific growth rate (SGR), and increase the utilization of feed. Furthermore, the candidate probiotic mixture had the ability to protect against NNV, which could decrease the mortality rate by 100% in giant grouper after NNV challenge. Subsequently, we analyzed the mechanism of the candidate probiotic mixture’s defense against NNV. A volcano plot revealed 203 (control vs. NNV), 126 (NNV vs. probiotics − NNV), and 5 (control vs. probiotics − NNV) differentially expressed transcripts in intestinal tissue. Moreover, principal components analysis (PCA) and cluster analysis heatmap showed large differences among the three groups. Functional pathway analysis showed that the candidate probiotic mixture could induce the innate and adaptive immunity of the host to defend against virus pathogens. Therefore, we hope that potential candidate probiotics could be successfully applied to the industry to achieve sustainable aquaculture.

## 1. Introduction

The intestine is colonized by a wide variety of species of bacteria that are considered the first line of defense against pathogens. These bacteria have multiple functions, such as maintaining intestinal homeostasis, developing the immune system, and maintaining homeostasis of the gut-brain axis [1,2]. Many studies in recent years have indicated that diseases might be associated with dysbiosis of the intestinal microbiota. Hence, the close linkage among gut microbiota, health, and disease has led to a new horizon in using probiotics to maintain the microbial balance in the intestine during pathogen infection [1]. Traditionally, antibiotics have been applied to treat bacterial infections for past 50 years in the aquaculture industry [3]. The indiscriminate use of chemicals will accelerate antibiotic resistance and transform bacteria into superbugs [4]. Accordingly, many countries have been emphasizing antibiotic resistance research, and health agencies have launched regulations encouraging the prudent use of antibiotics to limit their overuse [5].

Antibiotics do not work on viral infections; the treatments for viral infections are vaccines or antiviral drugs. Thus, modulation of the intestinal microbiota with “good” bacterial species is particularly well-known in animal disease treatment, especially in the aquaculture industry. Several probiotic treatments have been applied in a broad range of livestock animals over the past decades and are now becoming increasingly popular “magic drugs” in the livestock industry [6]. Probiotics refer to live microorganisms that have a positive effect on the health of the host when administered in adequate quantities and maintain the richness and diversity of the intestinal microbiota [7,8]. *Lactobacillus* and *Bacillus* are probiotics widely used in aquaculture, but several other genera, such as *Aeromonas, Alteromonas, Arthrobacter, Bifidobacterium, Clostridium, Paenibacillus, Phaeobacter, Pseudoalteromonas, Pseudomonas, Rhodosporidium, Roseobacter, Streptomyces* and *Vibrio*, are reported to enhance growth performance, maintain homeostasis of the intestine, and enhance disease resistance [9].

Recently, increasing evidence suggests the existence of communication between the intestine and the central nervous system (microbiota-gut-brain crosstalk) [10]. Intestinal disorders will influence the behavior and immune system of the host; for example, chronic intestinal inflammation may contribute to a higher risk of developing diseased individuals than healthy individuals [11,12].

Coincidentally, nervous necrosis virus (NNV) is also known as viral encephalopathy and retinopathy. Fish infected with NNV display neurological disorders such as abnormal swimming behavior, lack of appetite and lethargy [13]. NNV, which particularly affects fragile larval stages, is a tricky and highly problematic pathogen in the grouper industry [14]. However, no previous studies have evaluated the effect of probiotic treatment on NNV-infected grouper. Therefore, this study aimed to develop a novel potential complex probiotic strategy for NNV treatment in grouper. We isolated three strains of bacteria as candidate probiotics from healthy grouper’s intestines, and the growth parameters were assessed after dietary treatment with these candidate probiotics for 28 days. Through transcriptome analysis we can achieve a better understanding of the candidate probiotics’ mechanism of actions toward the prevention and treatment to NNV. These preliminary research results on these novel candidate probiotic strains could be expanded to develop new therapeutics for the treatment of viral disease to achieve long-term sustainability in the aquaculture industry.

## 2. Materials and Methods

### 2.1. Isolation of Potential Candidate Probiotics

Five healthy *Epinephelus lanceolatus* (50 ± 0.3 g) from hatcheries at Pingtung, Taiwan were sacrificed for sampling after anesthesia with 200 ppm 2-Phenoxyethanol. The whole intestine was dissected out and cut open under sterile conditions. First, intestinal content was removed with a spatula, providing respective samples of loosely associated bacteria [15]. After dissection, intestinal samples were washed twice with PBS-EDTA and immediately stored at −80 °C until subsequent use. To make an initial dilution (10^−1^), 100 µL of intestine mixture was homogenized with 900 µL of 0.9% sterile saline water. Two hundred microliters of these dilutions were pour-plated on two nonselective (tryptic soy agar (TSA) and brain heart infusion (BHI)) agar plates and incubated at 28 °C under anaerobic conditions (using anaerocult A gas packs; Merck) for 48 h. After 48 h, different individual colonies were phenotypically selected (different shape, size, colony morphology) and subcultured in tryptic soy broth (TSB) and BHI broth under anaerobic conditions for 48 h at 28 °C. In addition, whole intestines were isolated from three healthy individuals and placed into 10 cm Petri dishes containing sterile PBS on ice. Intestines were dissected and opened longitudinally, the intestinal contents were scraped out, and then the tissues were cut into 0.5 cm pieces to facilitate the release of bacteria. The collected intestinal contents were washed once in cold PBS and added to TSB at 28 °C for 48 h under anaerobic conditions. Glycerol stocks (50% *v*/*v*) were prepared for each colony and stored at −80 °C.

### 2.2. Identification of Isolates

Bacterial genomic DNA was extracted from liquid cultures with a Geneaid Genomic DNA Mini Kit, followed by PCR amplification with the universal eubacteria primers 27F and 1492R [16] in a final volume of 20 μL. Each 20-μL PCR mixture consisted of genomic DNA (50 ng), 10 μL of 2× Ready to Load PCR Master Mix (Cyrus Bioscience, MDBio. Inc., Annapolis, MD, USA), 0.5 μL of forward primer (10 µM), and 0.5 μL of reverse primer (10 µM). PCR was performed in a TProfesional Thermocycler^®^ (Analytik Jena AG, Jena, Germany) with the following cycling conditions: an initial denaturation at 95 °C for 2 min, 35 cycles of denaturation at 94 °C for 1 min, annealing at 55 °C for 1 min, and extension at 72 °C for 1 min. A final extension was performed at 72 °C for 10 min. PCR products were analyzed using 1% agarose gel electrophoresis. The size of the primer-amplified segment was expected to be 1465 bp. DNA fragments were purified using the GenepHlow™ Gel/PCR Kit (Geneaid) according to the manufacturer’s instructions. Sequencing of the PCR product was performed by Protech Technology Enterprise Co., Ltd. (Taipei, Taiwan). All the sequencing results were compared online with standard 16S rRNA sequences of bacteria in GenBank using nucleotide BLAST to identify close relatives.

### 2.3. Determination of the Growth Profile of the Candidate Probiotics

Three selected isolates (*Bacillus cereus* ATCC 14579, *Paraburkholderia fungorum* strain 2671 and *Enterobacter ludwigii* strain ED4) which were isolated from TSA plate were inoculated in 100 mL of TSB containing 1.5% NaCl under anaerobic conditions at 28 °C. The growth curves were measured for optical density at 600 nm using a UV–visible spectrophotometer (Genequant™ 100). The experiment was performed in triplicate for each candidate probiotic (Supplementary Figure S2). Preparation of candidate probiotic mixtures was carried out by inoculating the isolates in TSB for 8–9 h at 28 °C. The final concentrations of the three candidate probiotic isolates were adjusted to 10^9^ CFU/mL and mixed together.

### 2.4. Maintenance of Grouper

In this study, juveniles of *E. lanceolatus* with average body weight of 22 g ± 3 were collected from hatcheries at Pingtung, Taiwan. Grouper were cultured in environmentally controlled indoor facilities with a recirculating system (mechanical filter, biological filter, pump tank and pump) where all the groupers were under observation in a 2-ton fiberglass tank for two weeks. The flow rate (approximately 100 GPM) remained constant until the end of the trial. Standard environmental conditions were artificially established, such as aerators, heaters, and biofilters. Additionally, digital thermometers were connected to the tank to monitor the water temperature and maintain it at 30 °C. Fish were fed twice per day with commercial feed.

### 2.5. Feeding Trial

**Figure 1:**
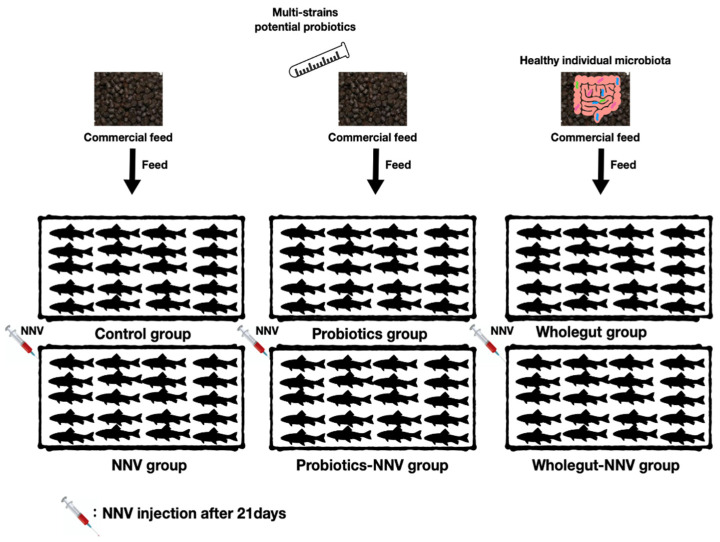
Graphical experimental design. Control and NNV groups were fed a commercial diet without any probiotics. Probiotics and probiotics-NNV (P.NNV) groups were fed commercial diet with mixture of the three potential probiotics (*B. cereus* ATCC 14579, *P. fungorum* strain 2671 and *E. ludwigii* strain ED4) (1:1:1109 CFU/mL, 10:1). The microbial transfer diet groups were called the wholegut and wholegut-NNV (W.NNV) groups. The feeding trial lasted for 28 days in triplicate (n = 20). By day 21, NNV challenge tests were performed in NNV, P.NNV and W.NNV and recorded daily 7 days after infection (dpi).

All experiments were conducted following National Taiwan Ocean University animal ethics guidelines (Approval number: 109014). The grouper were randomly separated into six experimental groups, which contained twenty animals per group in triplicate. The experimental design is shown in Figure 1. Control and NNV groups were fed a commercial diet without any probiotics which purchased from Taisun enterprise Co., Ltd. (Taipei, Taiwan). The commercial feed contains 45–48% crude protein, 4–5.5% fat, 2–3% fiber, 14–16% ash, 1.5–3% phosphorus, and 8–11% moisture. The three potential probiotics (*B. cereus* ATCC 14579, *P. fungorum* strain 2671 and *E. ludwigii* strain ED4) were mixed together (1:1:1, 10^9^ CFU/mL) and provided as a top dressing on the commercial diet (10:1) before being fed to the probiotics and probiotics − NNV (P.NNV) groups for 28 days. The microbial transfer diet groups were called the wholegut and wholegut-NNV (W.NNV) groups. The commercial feed was supplemented with the wholegut mixture (10^9^ CFU/mL), where the fish were regularly fed for 28 days. Feeding was performed twice daily. The feeding amount was adjusted every week at 5% of fish body weight.

### 2.6. Cell and Virus Preparation

The grouper fin cell line (GF-1) was used in this study [17]. Cells were maintained in Leibovitz’s L-15 medium (Gibco, USA) supplemented with 5% fetal bovine serum (BSA), 100 U/mL penicillin and 0.1 mg/mL streptomycin (MD Bio Inc. (Rockville, MD, USA)) at 28 ℃. RGNNV was originally isolated from brain tissue of *Epinephelus* spp. and propagated in GF-1 cells cultured in 2% FBS-supplemented L-15 medium at 28 °C. The virus titer of RGNNV (first passage) was determined to be 10^8^ TCID_50_/mL using the end-point method [18].

### 2.7. NNV Challenge Tests

Challenge tests were performed using the experimental groups, which were defined as the (1) NNV; (2) probiotics-NNV; and (3) wholegut-NNV groups after 21 days of the feeding trial. Infection was performed via intramuscular injection with 100 μL of NNV (1 × 10^8^ TCID_50_/mL) in triplicate per condition (*n* = 20) and individuals were continuously fed with a basal diet (control and NNV group) or diet containing bacteria (P.NNV and W.NNV group). The dead fish in each group were collected and recorded daily 7 days after infection (dpi). Intestinal, splenic, eye and brain tissues were collected immediately and kept at −80 °C until further analysis.

### 2.8. Evaluation of Growth Performance and Health Condition

Grouper weights were measured at the initial, 2nd, 3rd and final weeks of the experiment after anesthesia with 200 ppm 2-Phenoxyethanol. After the trial was completed, the growth parameters were estimated using the following standard equations: weight gain, WG (g) = Final body weight-Initial body weight (g), specific growth rate, SGR (%) = (ln (Final body weight) − ln (Initial body weight)/Day) × 100%, and feed intake, FI: (Total weight of feed (g))/(Weight of fish (g)). In addition, the cumulative mortality rate of the grouper was calculated after 7 dpi. Three independent replicates (*n* = 20) were performed in the survival group. Cumulative mortality rate = (Total deaths to NNV)/(Total of fish) × 100%.

### 2.9. Effects of Different Treatments with Potential Probiotics on the Expression of Immune Genes

Seven days post infection, five fish from each group were randomly sacrificed upon anesthetization with 100 ppm 2-Phenoxyethanol. The viscera were dissected in an ice box, and the whole intestine was gently cut and mixed together. The brain, eyes, head, kidney and spleen were also collected and stored in 1.5-mL Eppendorf tubes with 700 µL of TRIzol (Life Technologies). All the prepared samples were preserved at −80 °C until further analysis.

### 2.10. Total RNA Extraction, cDNA Synthesis and Determination of Immunological Genes

Total RNA from the grouper was extracted using TRIzol (Life Technologies) according to the manufacturer’s protocol. After RNA extraction, 1 µg of total RNA was used for cDNA synthesis with HiScript I Reverse Transcriptase (BIONOVAS, Toronto, ON, Canada) according to the manufacturer’s protocol. Reverse transcription was conducted according to the manufacturer’s protocol with random primers. The cDNA synthesis conditions were set at 65 °C for 5 min, 42 °C for 60 min and 70 °C for 15 min. Quantitative real-time PCR (qPCR) was performed using a TOptical Thermocycler^®^ (Analytik Jena AG, Jena, Germany). Each qPCR contained 1 μL of the cDNA template, 10 μL of 2X qPCRBIO syGreen Master Mix, and 0.8 μL each of the forward and reverse primers (10 pmol/µL) of a particular immune gene (Table 1). The amplification conditions were initial denaturation at 95 °C for 5 min, followed by 40 cycles of 95 °C for 5 s and 65 °C for 30 s. 

The melting curve and cooling were performed in the last step of qPCR. The expression levels of the immune-related genes were normalized to beta-actin, a housekeeping gene. The fold change in the relative gene expression of the control group was determined by the standard 2^−^^△△^Ct method. The changes were analyzed by unpaired sample *t*-tests. Statistical significance was accepted at *p* < 0.05, and high significance was accepted at *p* < 0.01. All data are expressed as the mean ± standard deviation (mean ± SD).

### 2.11. Transcriptome Analysis

Total grouper RNA was extracted using TRIzol (Life Technologies) according to the manufacturer’s protocol. RNase-free DNase I (GMbiolab, Taiwan) was used to remove the residual genomic DNA. Then, the RNA concentration and quality were verified with an Agilent 2100 Bioanalyzer. The extracted RNA was stored at −80 °C until it was processed for library preparation. After RNA extraction, RNA was quantified to 150 ng/µL and total RNA was fixed at 20 μg. Transcriptome sequencing was then carried out as a commercial service at Genomics Biotechnology Co., Ltd., Taiwan, on an Illumina HiSeq™ 2000. The attached adaptor sequences on reads were removed. Reads containing more than 45% low-quality (<Q20) bases were discarded. After the QC process, the reads were de novo assembled using Trinity v2.2.0 software. Assembly was completed in an Ubuntu 16.04 based environment. Differentially expressed transcripts were identified by running the script run_DE_analysis.pl, and the differential expression analysis method was edgeR (version 4.1, accessed on 18 May 2021) based on the R package. The differentially expressed genes (DEGs) showing expression levels with fold-change absolute values of more than 1 and FDR (false discovery rate) p values of less than 0.001 were considered significant and were collected for the following analysis. The Blast2ref tool was used for functional annotation to investigate the biological functions of the DEGs.

## 3. Results

### 3.1. Identification of Isolated Bacteria

Initially, 15 morphologically distinct bacteria isolated from the intestinal tract of five healthy groupers were collected (Supplementary Figure S1). The PCR products of the isolated bacteria were verified by gel electrophoresis (1465 bp) with the universal eubacteria primers 27F and 1492R before gene sequencing. Among the isolates, *Bacillus cereus* ATCC 14579, *Paraburkholderia fungorum* strain 2671, and *Enterobacter ludwigii strain* ED4 were used in this study.

### 3.2. Candidate Probiotics Influence the Growth of E. lanceolatus

Experimental diets were prepared as described previously. The growth performance parameters assessed in this study included initial body weight, final body weight, weight gain (WG), specific growth rate (SGR) and feed intake (FE). Table 2 shows the growth performance after dietary intake of candidate probiotics. The WG, SGR and FE were increased in the probiotic group compared with the control group after 28 days. However, all growth performance parameters of the whole gut group were lower than those of the control and probiotic groups. Dietary treatment with three strains of candidate probiotics could improve growth performance in giant grouper.

### 3.3. Candidate Probiotics Elicit Great Protection after NNV Challenge

**Figure 2:**
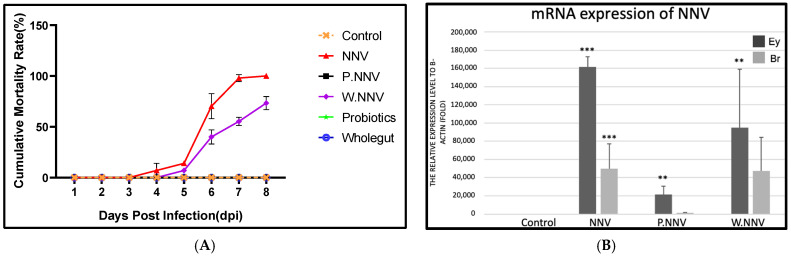
The NNV challenge test 21 days after probiotic diet feeding. (**A**) The cumulative mortality rate after NNV challenge. Giant grouper were treated with different diets for 28 days. 21 days post administration, the giant groupers were challenged with NNV (100 µL of 10^5.5^ TCID_50_/mL per fish). Control: commercial feed + no NNV challenge; NNV: commercial feed + NNV challenge; W.NNV: commercial feed top dressing with microbial transfer mixture + NNV injection; P.NNV: commercial feed top dressing with candidate probiotic mixtures + NNV challenge. Experiments were performed in triplicate (*n* = 20). Fish mortality was recorded daily. (**B**) The expression of NNV in eye (Ey) and brain (Br) tissues. The tissues were collected randomly from the dead grouper (*n* = 5) in triplicate. All treatment groups were compared to the control diet group with significant differences shown as: ** *p* < 0.01 and *** *p* < 0.001.

In the NNV challenge test, giant grouper were fed the experimental diet for 28 days, and Figure 2A presents the cumulative mortality rate of NNV-infected giant grouper at 7 days post infection (dpi).

All giant grouper in the control group remained healthy and survived until 7 dpi. Mortality in the NNV group started at 3 dpi and reached a cumulative mortality rate of 100% at 7 dpi. Furthermore, the cumulative mortality reached in the W.NNV group was 74% by 7 dpi. The diseased grouper displayed several clinical signs, including whirling and spiraling. Interestingly, no mortality or clinical signs were observed in the P.NNV group during the challenge test. The qPCR tests performed randomly on dead grouper demonstrated that mortality was caused by NNV. As shown in Figure 2B, the results showed that NNV mRNA expression in the NNV and W.NNV group was significantly increased in both the eye and brain tissues, which was comparable to the P.NNV group, and no viral expression was detected in the control group. Through these data, the results indicated that the three strains of candidate probiotics could enhance NNV resistance in giant grouper.

### 3.4. Identification of Genes Differentially Expressed in the Intestines of E. Lanceolatus after Dietary Probiotic Treatment

**Figure 3:**
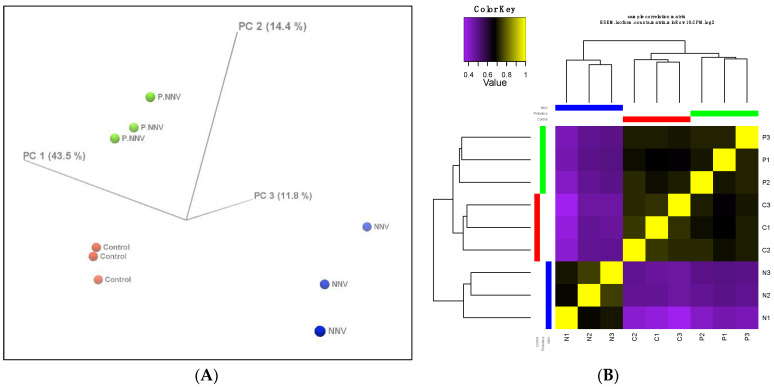
Profiles of differentially expressed genes. (**A**) 3D PCA scatter plot. Characteristics of the control, probiotics − NNV (probiotic) and NNV groups according to the gene expression profiles. Each dot indicates a sample. (**B**) Hierarchical clustered expression analysis comparing the RNA-seq patterns of the control (C), NNV (N) and probiotics − NNV (P) groups. Yellow indicates higher expression, and purple indicates lower expression.

The differentially expressed genes between the control, NNV and probiotics − NNV groups were determined. The gene expression profiles were evaluated by principal components analysis (PCA) to provide insights into the associations among the control, NNV and probiotics − NNV groups. Figure 3A shows clear clusters among the three groups. Moreover, the control and probiotics − NNV groups showed large differences from the NNV group. We further performed hierarchical clustering analysis of the gene expression profiles among the individuals from the three groups. From the cluster analysis heatmap (Figure 3B), we determined that the expression patterns of the control and probiotics − NNV groups shared higher similarity than those of the NNV group.

**Figure 4:**
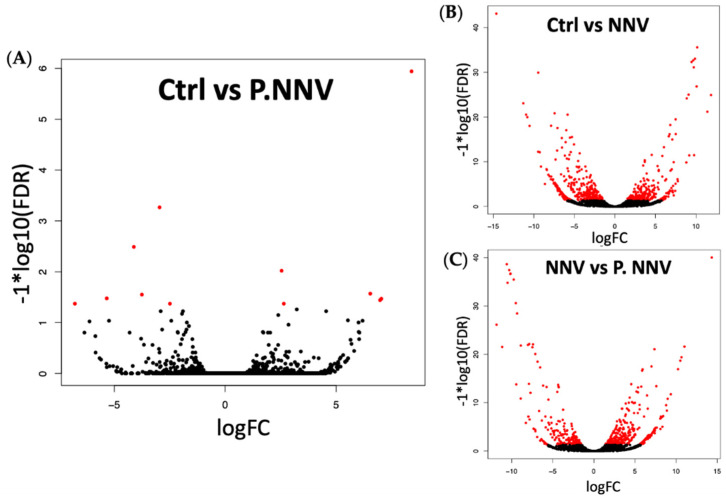
Volcano plot of differential gene expression. Grouper treated with (**A**) candidate probiotics + NNV infection vs. untreated grouper as controls; (**B**) NNV vs. untreated grouper as controls; (**C**) candidate probiotics + NNV infection vs. NNV-treated grouper. Scattered points represent genes: the x-axis is the log 2-fold change for the ratio, whereas the y-axis is the probability that a gene has statistical significance in its differential expression. Red dots are the genes significantly overexpressed after treatment, and black dots are the genes significantly underregulated after treatment.

Cluster analysis indicated that the gene expression profile of the probiotics − NNV group was highly related to the control group. To identify the significant differentially expressed genes in the transcriptome, the control, NNV and probiotics − NNV groups were compared. A fold-change absolute value greater than 1 and an FDR p value less than 0.001 were considered significant. Pairwise comparisons between the two experimental conditions separated the results into three parts: control vs. probiotics − NNV (Figure 4A), control vs. NNV (Figure 4B), and NNV vs. probiotics − NNV (Figure 4C). The results of the RNA-Seq analysis revealed 203 (control vs. NNV), 126 (NNV vs. probiotics − NNV), and 5 (control vs. probiotics − NNV) differentially expressed transcripts in intestinal tissue after dietary administration of probiotics.

### 3.5. Pathways Enriched in the Intestines of E. Lanceolatus after NNV Challenge

**Figure 5:**
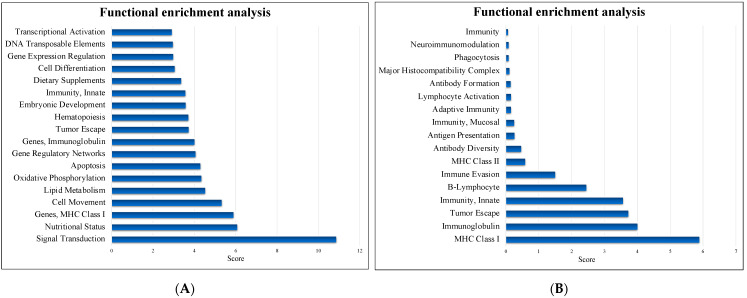
Functional enrichment analysis. (**A**) The most enriched pathways and (**B**) the immune system pathways identified by Blast2fish (http://blast2fish.ntou.edu.tw, accessed on 15 September 2021). Pathways were upregulated in the probiotics − NNV group vs. the NNV group.

The DEGs were mapped to BLAST2Fish for functional enrichment analysis of nonmodel teleost fish transcriptome data. The DEGs were primarily enriched in signal transduction, nutritional status and MHC class I (Figure 5A). Among the immune systems, a total of 329 genes were DEGs in the intestine after treatment with NNV and P.NNV (Figure 5B), in categories such as adaptive immune response (MHC class I and immunoglobulin) and innate immunity.

**Figure 6:**
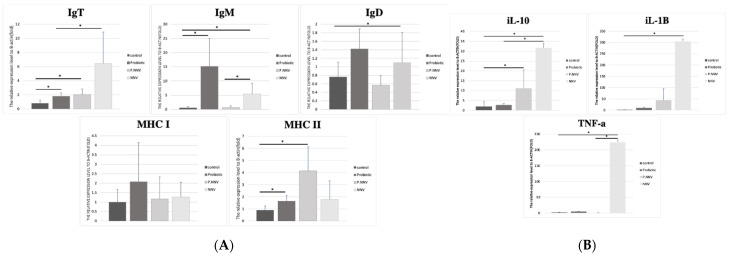
(**A**) Adaptive and (**B**) innate immune-related gene expression levels in the intestine of giant grouper after treatment with probiotics and infection with NNV. The expression levels of immunoglobulins (IgT, IgM and IgD), major histocompatibility complex (MHC I and MHC II) and inflammatory factors (TNF-*α*, IL-10 and IL-1*β*) in the intestine. Control: untreated group; NNV: NNV infection group; P.NNV: probiotics − NNV group; Probiotics: probiotics-fed group. The relative mRNA levels were normalized to *β*-actin. Data presented are presented as the mean ± SD (*n* = 5), and the asterisks (*) represent significant differences at * *p* < 0.05.

The sequenced RNA samples and immune organs were validated by qPCR for adaptive and innate immune responses. As shown in Figure 6A, the upregulations of IgT in the probiotic and P.NNV groups were observed to be significantly higher than those of the control group, while the NNV group presented higher expression than the other groups. In addition, the IgM and IgD expression levels in the probiotic and NNV groups were significantly higher than those in the control group, whereas the expression in the P.NNV group was downregulated compared to the NNV and probiotic groups. As shown in Figure 6B, infection with NNV caused an increase in the innate immune response in intestinal tissues. In the intestines, the IL-10 and IL-1*β* expression levels of the probiotic group were higher than those of the control group.

**Figure 7:**
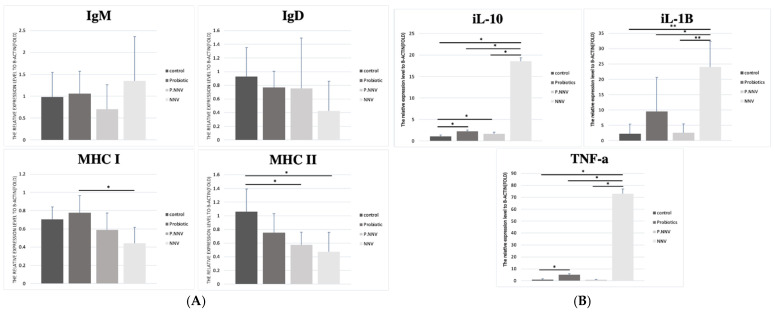
(**A**) Adaptive and (**B**) innate immune-related gene expression levels in the spleen of giant grouper after treatment with probiotics and infection with NNV. The expression levels of immunoglobulins (IgT, IgM and IgD), major histocompatibility complex (MHC I and MHC II) and inflammatory factors (TNF-*α*, IL-10 and IL-1*β*) in the spleen. Control: untreated group; NNV: NNV infection group; P.NNV: probiotics − NNV group; Probiotics: probiotics-fed group. The relative mRNA levels were normalized to *β*-actin. Data presented are presented as the mean ± SD (*n* = 5), and the asterisks (*) represent significant differences at * *p* < 0.05 and ** *p* < 0.01.

A higher induction of MHC I was observed in the intestines of the probiotic group, similar to the spleen. The MHC II expression levels in the P.NNV and probiotic groups were significantly higher than the control group. In the spleen (Figure 7A), MHC II expression in the treatment groups (probiotic, P.NNV and NNV) was downregulated compared with the control group. As shown in Figure 7B, treatment with NNV caused an increase in the innate immune response in spleen tissues. The expression levels of IL-1*β*, IL10 and TNF-*α* (spleen) in the probiotic group were slightly higher than those in the control group. In both the intestine and the spleen, innate immune gene expression (IL-10, IL-1β and TNFα) was induced after NNV infection. Overall, probiotic dietary treatment may trigger both innate and adaptive immune genes to inhibit viral pathogens and inhibit the inflammatory response in the intestine and spleen.

## 4. Discussion

Probiotics refer to a combination of live microorganisms that benefit the host’s health when consumed in adequate amounts [19]. Previous studies have revealed that numerous probiotics have the potential to become promising alternatives to antibiotics and chemical compounds [20,21]. Increasing research has demonstrated that multiple strains of probiotics could be more effective for maintaining homeostasis of the intestinal tract and enhancing disease resistance in animals [22,23,24,25]. It has long been known that many commercial probiotics are derived from humans or terrestrial animals and act as feed additive to aquatic animals. Therefore, there is limited ability to control the growth of harmful microorganisms in aquatic environments. We expected native probiotics isolated from the original host to achieve the greatest effect on aquatic animals and dependently [24,25]. In this study, we isolated three strains of candidate probiotics from *E. coioides* intestines, *Bacillus cereus* ATCC 14579, *Paraburkholderia fungorum* strain 2671, and *Enterobacter ludwigii* strain ED4, and characterized their effectiveness in controlling viral disease. *B. cereus* ATCC 14579 has the characteristic of synthesizing bacteriocin-like substances during growth and is able to inhibit the growth of pathogen-related bacteria such as *Staphylococcus aureus* and *Micrococcus luteus* [26]. A previous study reported that *B. cereus* could be a biological additive reagent to improve water quality, growth rate and immune response in cultivated shrimp [27]. Another candidate probiotic, *P. fungorum*, is a plant probiotic that can increase the growth and production of strawberries. *P. fungorum* is a novel microbiome-based plant breeding strategy that can regulate growth and disease defense [28]. It is known that *E. ludwigii* is a candidate probiotic in aquaculture, as it enhances the digestive enzyme activity of *Haliotis asinina* L. In addition, *E. ludwigii* isolated from *Aedes albopictus* has been shown to inhibit La Crosse encephalitis, suggesting that the bacteria may secrete antiviral metabolites [29,30]. Ideally, these candidate probiotics have unique functions in different aspects, such as improvement of the immune status, digestive process, growth performance and viral and bacterial infection resistance; thus, the efficacy of these candidate probiotics was evaluated in grouper.

The involvement of probiotics in nutrition, growth performance and other beneficial activities in aquatic animals has been proven beyond any doubt [31]. Several studies have suggested that probiotics can improve the growth condition of aquatic animals. For example, in tilapia, the specific growth rate and feed intake increased after administration of *Clostridium butyricum* for 56 days [32]. An Iranian team demonstrated significant increases in the condition and final body weight of common carp after treatment with mixtures of *Lactobacillus spp.*, *Bifidobacterium bifidum*, *Streptococcus silivarius*, *Enterococcus faecium*, *Aspergillusoryzae* and *Candida pintolopepsii*. Similarly, better growth performances were observed in white shrimp with dietary administration of *L. fermentum*, *L. pentosus*, *Saccharomyces cerevisiae* and *B. subtilis*, resulting from significantly improved feed efficiencies [24]. Probiotics are broadly considered as safe dietary supplements. However, there are some risks increased if the host’s immune system is weakened or has other serious chronic conditions, including the development of other disease infection, resistance to antibiotics and developing harmful by products [33]. Based on our results (Table 2), we consider that the candidate probiotic mixture was able to improve the digestive health and strengthen the growth performance of giant grouper.

In the aquaculture industry, viral infections are always a major concern since they can cause massive economic losses worldwide. Currently, many studies have suggested that probiotics have the capacity for disease resistance by enhancing the host immunity [34]. There are many types of probiotics that have different effects, such as the production of inhibitory compounds (such as bacteriocins and aflatoxins), competition for active localization of sites to prevent opportunistic or pathogenic microorganisms, competition for nutrients with other bacteria or an improvement of the host’s immune system [35,36]. In our study, administration of a diet with a candidate probiotic mixture achieved a good effect in controlling NNV in giant grouper (Figure 2). In contrast, there was only a 26% reduction in mortality rate in the giant grouper treated with microbial transfer therapy from NNV infection. Studies have shown a significantly reduced mortality rate of iridovirus-infected grouper after dietary administration of lactic acid bacteria [37]. Ramasamy Harikrishnan et al. stated that administration of *Lactobacillus spp.* for four weeks could increase the survival rate of olive flounder infected by LCDV. The effectiveness of probiotics is dependent on the successful establishment of probiotics in the gut. The secretion system (Type III or VI) of *P. fungorum* is highly responsible for survival, adhesion, and adaptation [38]. The type III secretion system is a nanomachine that delivers effector proteins into the host cell to mediate pathogenesis [38], and type VI is a toxin delivery system which antagonizes competitors [39]. As we mentioned above, *E. ludwigii* may secrete antiviral metabolites; however, the discovery of promising antiviral compounds is still novel [40]. PlcR, a transcriptional regulator of *B. cereus* strain ATCC14579, was reported to repress biofilm formation [41]. NprR was disrupted by a transposon in *B. cereus* strain ATCC14579, which led to the shutdown of the necrotrophic regulon, which means the NprR dominate the necrotrophic characteristics allowing *B. cereus* strain ATCC14579 to survive in intestine of the infected host.

A previous study evaluated the intestinal transcriptome profile of zebrafish after immersion with multiple *Lactobacillus* strains from hatching to the adult stage. Exposure to *L. casei* BL23 treatment for 35 days revealed 369 DEGs in the intestine that involved several growth factors, such as signaling, secretion, motor proteins, tight junctions, lipid metabolism, growth regulation, proteases and humoral and cellular effectors [42]. To the best of our knowledge, there have been no reports on transcriptome analysis after dietary treatment with multistrain probiotics on grouper. From our results (Figure 5), these findings suggested that the immune responses of giant grouper were enriched in the intestines after NNV infection, indicating a positive correlation between these candidate probiotics and immunity.

Based on the RNA-seq data (Figure 6), the functional pathways mainly included MHC, immunoglobulins and innate immunity. In our study, innate immune gene expression (IL-10, IL-1*β* and TNF*α*) was induced in both the intestine and the spleen after NNV infection. However, there were no changes in innate immune gene expression in the probiotic and P.NNV feeding groups. A previous study revealed the high expression of proinflammatory cytokines (IL-1*β* and TNF*α*) after NNV infection in grouper [43]. Many studies have shown that NNV infection is highly related to the inflammatory response and that inflammatory cytokines play an important role in NNV inhibition [44,45,46]. We suggest that probiotics might have the potential to inhibit NNV activity, as suggested by P.NNV, which had lower expression of IL-1*β* and TNF*α* than NNV in both intestinal and splenic tissues. In teleosts, B cells are composed of three antigen-specific immunoglobulins: IgM, IgD, and teleost-specific IgT [47,48,49]. Immunoglobulins play an important role in the adaptive immune system to protect fish from pathogen infection in aquatic environments [50,51]. IgT, like IgA in mammals, plays a vital role in mucosal immunity and might modulate the humoral immune response driven by the intestinal microbiome [52]. In this study, we determined that the expression level of IgT was significantly increased in the NNV infection group but was decreased in the probiotic and P.NNV groups (Figure 6A), indicating that a positive correlation occurred: the higher the viral expression of NNV in grouper was, the higher the expression of IgT in grouper. This finding corresponded to a study on rainbow trout, showing lower levels of IgT and IgM B cells in healthy conditions in the lamina propria [53,54]. Recent findings suggested that IgT and IgM expression levels were upregulated upon pathogen infection of rainbow trout [55]. However, the contribution of IgD in the intestines remains unknown. Moreover, there was higher expression of MHC class II in the P.NNV group (Figure 6A), while there was no significant difference in MHC class I expression among the groups in the intestines. Thus, we implied that the fish immune response occurs through MHC class II during long-term feeding with probiotics. MHC class II molecules are present on antigen-presenting cells and can activate CD4 T cells, which express pathogen antigens to activate helper T cells [56]. Through these data, we hypothesized that antigens from probiotics might be taken up by antigen-presenting cells (MHC II) and presented to CD4 cells. Thereafter, IgT B cells were activated by Ag-specific CD4 T cells, indicating that both T- and B-cell responses were triggered by the antigen. In contrast, it is interesting to note that the effects in the P.NNV group may be due to long-term feeding with probiotics and may indicate their potential to provide protection from NNV infection through the T-cell response.

In summary, the present study underlines the efficacy of treatment with multistrain probiotics in enhancing growth performance and defending against virus infection by stimulating host immunity. For disease prevention and treatment, traditional therapeutics focus on antibiotic and chemical substance consumption [57]. However, the overuse of antibiotics has generated many unwanted side effects in the community [58]. The negative impact of excessive use has caused several problems, including antibiotic resistance and residues found in aquaculture. Therefore, the discovery of new strategies to improve disease problems is a major goal. To solve the problem of antibiotic overuse, many countries have formulated stringent regulations on the usage and dosage of antibiotics in cultured aquatic animals. In summary, we found that the potential probiotics have positive effect on growth performances of giant grouper and reduce the mortality rate from NNV infection by a decrease of 100%. Long-term feeding with candidate probiotics might induce the adaptive immunity of grouper to defense against NNV, especially the T-cell response. Supplementation with a novel strain of probiotics for preventing viral disease is an environmentally friendly approach to the sustainability of the aquaculture industry.

## Figures and Tables

**Table 1:** Primer sequence used in this study.

Primer Name	Orientation	Nucleotide Sequences (5′-3′)	Primer Usage
27F	sense	AGAGTTTGATCCTGGCTCAG	PCR
1492R	antisense	GGTTACCTTGTTACGACTT	
beta-actin	sense	TCCACCGCAAATGCTTCTAA	real-time PCR
	antisense	TGCGCCTGAGTGTGTATGA	
NNV	sense	TGTCGCTGGAGTGTTCG	
	antisense	GAAGTCATTTGTGGAAAGGGAATC	
IgD	sense	ATTTTgACgCCAAgTTgACC	
	antisense	TgCCAGCTTGAAAATGATG	
IgM	sense	CTATCTGCTGGGCAGGTgTT	
	antisense	GCAGCAGAATCTTCAGTCTTCA	
IgT	sense	TGTGTCAAAGTCTGCCTGGGATTCA	
	antisense	CTTAGGAGGTGGAGGAGGCTTTTG	
MHC I	sense	TCACAATGAAAGCCTGGATTTATCT	
	antisense	GGTTCTGCTCTCCTGGTGTTA	
MHC II	sense	GTTCAGCAGCAGTTTGGG	
	antisense	ACTTAGTCAGAGCAGCCT	
IL-1B	sense	CCAGCGTTGAGGGCAGAA	
	antisense	ATCGTCTCCAGATGTAAGGTT	
IL-10	sense	GGAGAGGCTCAGAGGAAG	
	antisense	ACACCTGAGTGTGAGAACAGTAA	
TNF-a	sense	GCAAAGCCTCGCTGATG	
	antisense	GCCCAGATAAATGGCGTTGT	

**Table 2:** Growth performance of giant grouper (*n* = 20) in triplicate per condition fed a control diet and candidate probiotic mixture (probiotics) diet and the transfer microbiota (wholegut) diet for 28 days. The asterisks (*) represent significant differences at * *p* < 0.05.

Items	Diet		
	Control Group	Probiotics Group	Wholegut Group
Initial body weight (g)	24.67 ± 0.3	25.2 ± 0.3	24.62 ± 0.3
Final body weight (g)	40.31 ± 1.6	44.37 ± 0.9	39.06 ± 1.4
Weight gain (g)	15.65 ± 1.4	19.17 ± 0.5 *	14.33 ± 1.0
SGR (%day − 1)	0.76 ± 0.02	0.88 ± 0.01	0.72 ± 0.02
FE (%)	0.1 ± 0.008	0.12 ± 0.002 *	0.09 ± 0.004

## Supplementary Materials

The following are available online at https://www.mdpi.com/article/10.3390/life11101053/s1, Supplementary Figure S1: Identification of isolated bacteria. Supplementary Figure S2. The procedure of candidate probiotics isolation.
