# Supplementary material for: Dietary Administration of Novel Multistrain Probiotics from Healthy Grouper Intestines Promotes the Intestinal Immune Response against NNV Infection
Source: Life (Basel). 2021 Oct 7;11(10):1053. doi: 10.3390/life11101053 (PMC8539657; doi:10.3390/life11101053)
Supplement: Supplementary file 1 [file life-11-01053-s001.zip › life-1404900-supplementary.pdf]

Supplementary Materials: Dietary Administration of Novel Multistrain Probiotics from Healthy Grouper Intestines Promotes the Intestinal Immune Response Against NNV Infection

Joan Tang Xiao Joe, Henry Tan Shi Sung, Jen-Leih Wu , Yu-Shen Lai and Ming-Wei Lu

(A)

1500bp

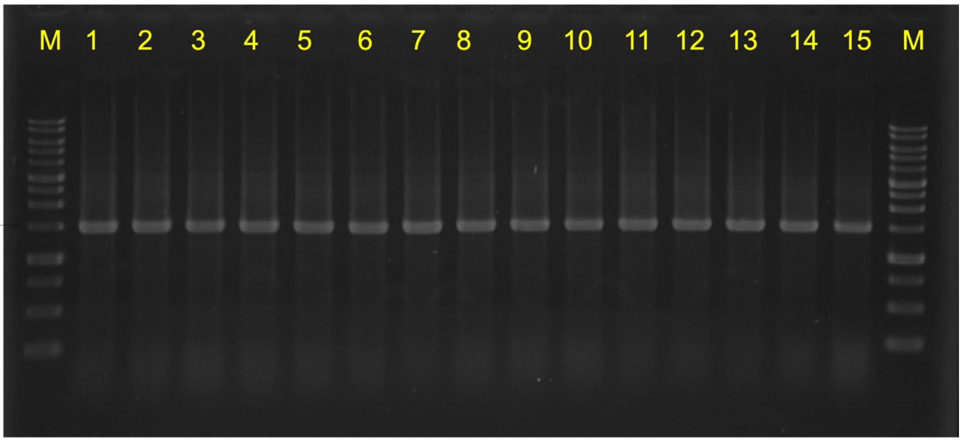

(B)

	Phylogenetic classification				BLAST analysis	
	Phylum	Class	Family	Closest relative	Accession no.	Similarity [%]
1	Unknown	Unknown	Unknown	Uncultured bacterium clone SW1112-21	KM269685.1	99.53
2	Unknown	Unknown	Unknown	Bacterium strain BS2065	MK825253.1	99.33
3	Gammaproteobacteria	Enterobacterales	Enterobacteriaceae	Klebsiella pneumoniae strain F16KP0064	CP052172.1	99.43
4	Gammaproteobacteria	Enterobacterales	Enterobacteriaceae	Enterobacter ludwigii strain ED4	MT613372.1	98.2
5	Betaproteobacteria	Burkholderiales	Burkholderiaceae	Paraburkholderia sp. strain LH5	MK920176.1	99.62
6	Gammaproteobacteria	Vibrionales	Vibrionaceae	Photobacterium damselae strain 9046-81	CP046752.1	99.03
7	Gammaproteobacteria	Vibrionales	Vibrionaceae	Vibrio sp. 785M	KU667051.1	98.47
8	cocci	Bacillales	Staphylococcaceae	Staphylococcus warneri strain HBU7252	MW355879.1	79.05
9	Unknown	Unknown	Unknown	Uncultured bacterium clone YZ05	KJ457330.1	99.43
10	Gammaproteobacteria	Vibrionales	Vibrionaceae	Vibrio sp. strain T3	MN049736.1	99.51
11	Gammaproteobacteria	Enterobacterales	Enterobacteriaceae	Enterobacter ludwigii strain JDM-11	JF690979.1	99.86
12	Bacilli	Caryophanales	Bacillaceae	Bacillus cereus ATCC 14579	NC004722.1	100
13	Gammaproteobacteria	Enterobacterales	Enterobacteriaceae	Klebsiella sp. STFXR7	LC484662.1	100
14	Gammaproteobacteria	Enterobacterales	Enterobacteriaceae	Enterobacter sp. NA-Z-FJ	KF358287.1	99.93
15	Betaproteobacteria	Burkholderiales	Burkholderiaceae	Paraburkholderia fungorum strain 2671	MT611690.1	98

Supplementary Figure S1. Identification of isolated bacteria. (**A**) Agarose gel electrophoresis of bacterial isolates PCR products. Lane M: 1.5 Kb DNA ladder, lanes 1-15: bacterial isolates PCR products (1465 bp). (**B**) Molecular identification of isolates based on partial 16S rRNA gene sequence information.

(A)

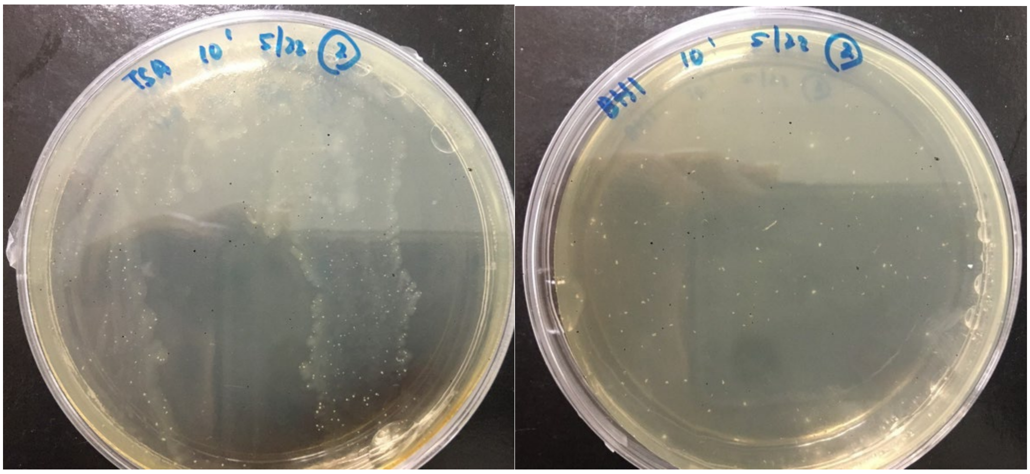

(B)

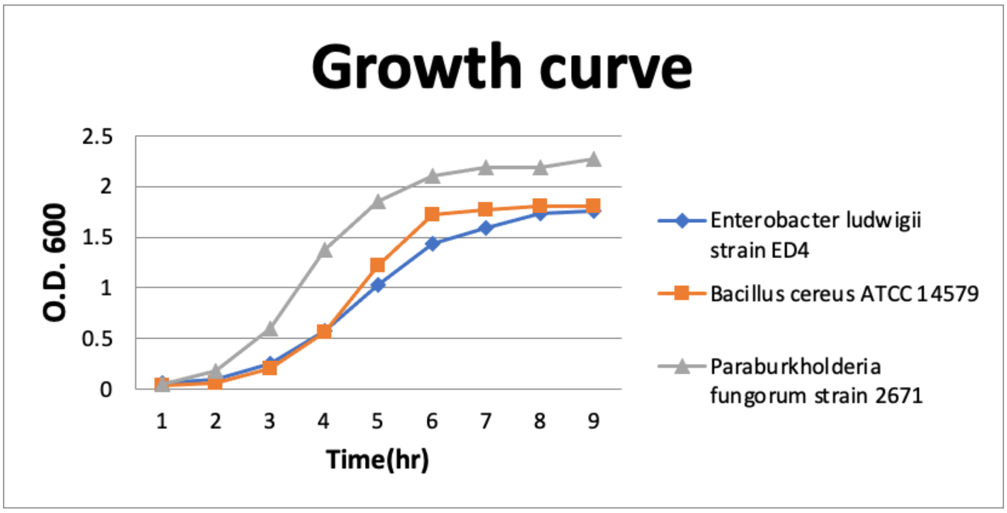

Supplementary Figure S2. The procedure of candidate probiotics isolation. (**A**) The intestine mixture was pour-plated on two non-selective (TSA and BHI) agar and incubated at 28 °C with anaerobic condition (using anaerocult A gas packs; Merck) (**B**) The Growth curve of Bacillus cereus ATCC 14579, Paraburkholderia fungorum strain 2671, and Enterobacter ludwigii strain ED4. The OD600 values were monitored for each culture with a 1 h interval. Experiments were done in triplicate.
